# Distribution and Diversity of Planktonic Fungi in the West Pacific Warm Pool

**DOI:** 10.1371/journal.pone.0101523

**Published:** 2014-07-03

**Authors:** Xin Wang, Purnima Singh, Zheng Gao, Xiaobo Zhang, Zackary I. Johnson, Guangyi Wang

**Affiliations:** 1 Department of Microbiology, University of Hawaii at Manoa, Honolulu, Hawaii, United States of America; 2 Collaborative Innovation Center of Deep Sea Biology, Tianjin University Center for Marine Environmental Ecology, School of Environment & Engineering, Tianjin University, Tianjin, China; 3 State Key Laboratory of Crop Biology, College of Life Sciences, Shandong Agricultural University, Tai'an, China; 4 Collaborative Innovation Center of Deep Sea Biology, College of Life Sciences, Zhejiang University, Hangzhou, China; 5 Marine Laboratory, Nicholas School of the Environment, Duke University, Durham, North Carolina, United States of America; 6 College of Engineering, California Baptist University, Riverside, California, United States of America; Instituto de Biologia, Brazil

## Abstract

Fungi contribute substantially to biogeochemical cycles of terrestrial and marine habitats by decomposing matter and recycling nutrients. Yet, the diversity of their planktonic forms in the open ocean is poorly described. In this study, culture-independent and molecular approaches were applied to investigate fungal diversity and abundance derived from samples collected from a broad swath of the Pacific Warm Pool across major environmental gradients Our results revealed that planktonic fungi were molecularly diverse and their diversity patterns were related to major phytoplankton taxa and various nutrients including nitrate, nitrite, orthophosphate and silicic acid. Over 400 fungal phylotypes were recovered across this region and nearly half of them grouped into two major fungal lineages of Ascomycota and Basidiomycota, whose abundance varied among stations. These results suggest that planktonic fungi are a diverse and integral component of the marine microbial community and should be included in future marine microbial ecosystem models.

## Introduction

The ‘microbial loop’ hypothesis depicts microbes as a central player of marine matter and energy fluxes [1]. Molecular taxonomy and ecological genomics have revealed the efficiency with which organic carbon is processed by marine microbes in the surface waters of the ocean [2]. Specifically, heterotrophic bacteria and archaea are found to be largely involved in carbon and nutrient cycling in both coastal and oceanic waters [2], [3]. Although heterotrophic eukaryotic microbes are well documented in the ocean, their diversity and function remain relatively unknown. Particularly, large populations of planktonic fungi (*i.e.*, filamentous free-living fungi and yeasts, and those associated with planktonic particles or phytoplankton) have long been known to exist in coastal and oceanic waters [4], [5], but their diversity and ecological function are still one of the most under-studied microbial topics.

Fungi are key components of the biosphere, performing a wide range of biogeochemical and ecological functions across disparate environments [6], [7], and are particularly well-known for their important role in processing detrital organic matters from plants [8]. Marine yeasts have long been known to be ubiquitous in seawater [9]–[11]. A typical milliliter of seawater contains several thousands of fungal cells (or propagules) [12]. In freshwater ecosystems, fungal biomass can account for as much as 18–23% of the total mass of detritus [14]–[16]. Recently, fungal mycelia were found to be present as individual filaments or aggregates in the coastal upwelling ecosystem off central Chile [17] and the Hawaiian coastal waters [13]. These filaments or aggregates can reach up to 20 mm in diameter and over 50 mm long and are comparable to fungal mycelia detected in deep-sea sediments [18] and water-stable aggregates associated with mycorrhizal fungi in soils [19]. In seawater, organic aggregates represent a major growth habitat for planktonic microbial communities. The combined metabolic activities of fungi and prokaryotic microbes can promote a highly efficient conversion of particulate organic matter (POM) to dissolved organic matter (DOM) in seawater [17], [20]. Furthermore, fungal biomass and diversity display spatial and temporal variations in the coastal marine ecosystems [5], [17]. During the summer, fungal biomass can reach up to ∼6 mg C/L of surface seawater [17] and the high fungal biomass was reported to be comparable to that of bacterioplankton in the coastal water [21]. The vertical profile of fungal biomass and diversity concurred with those of primary productivity and/or physical parameters of the water columns (e.g., temperature and oxygen) [5], [17]. Clearly, planktonic fungi are an important microbial component in the coastal marine ecosystems and, like other heterotrophic microplankton, are active in the water column and responding to primary production activity and organic matter availability [13], [17], [21].

In our previous study, we observed a novel diversity of planktonic fungi and their interesting relationship with biological and physical features of seawater in the Hawaiian coastal waters [5]. In this study, we focused on the diversity and ecology of mycoplanktonic communities in oceanic waters with the intention to gain insight into the following questions. What is the fungal diversity in the West Pacific Warm Pool? How is the fungal diversity and abundance partitioned in this oceanic ecosystem? Is a certain phylum of fungi dominant at a given location? How does the fungal diversity correlate with bacterioplankton and primary producers?

## Results and Discussion

### Fungal diversity and major phyla abundance in the open ocean

To assess the abundance and diversity of planktonic fungi in the open ocean, we collected seawater samples from five depths in the euphotic zone from stations across an open ocean transect from the Hawaiian coast to Australia (Figure S1 in File S1). A total of 959 clones were selected from 30 clone libraries of the internal transcribed region (ITS) region of the ribosomal RNA genes for sequencing analysis. Sequence analyses identified 411 distinct phylotypes (99% similarity) from 6 different stations (Tables S1 and S2 in File S1). Such a high diversity of planktonic fungi (and/or fungi like eukaryotes) was reported for the first time from the oceanic waters. Fungal diversity found in this study was much higher than that of previous report in Hawaiian coastal waters [5]. The diversity discrepancy clearly ascribed to differences in diversity analysis methods (clone library vs. DGGE), and primers used in those studies [7], [12].

**Figure 1:**
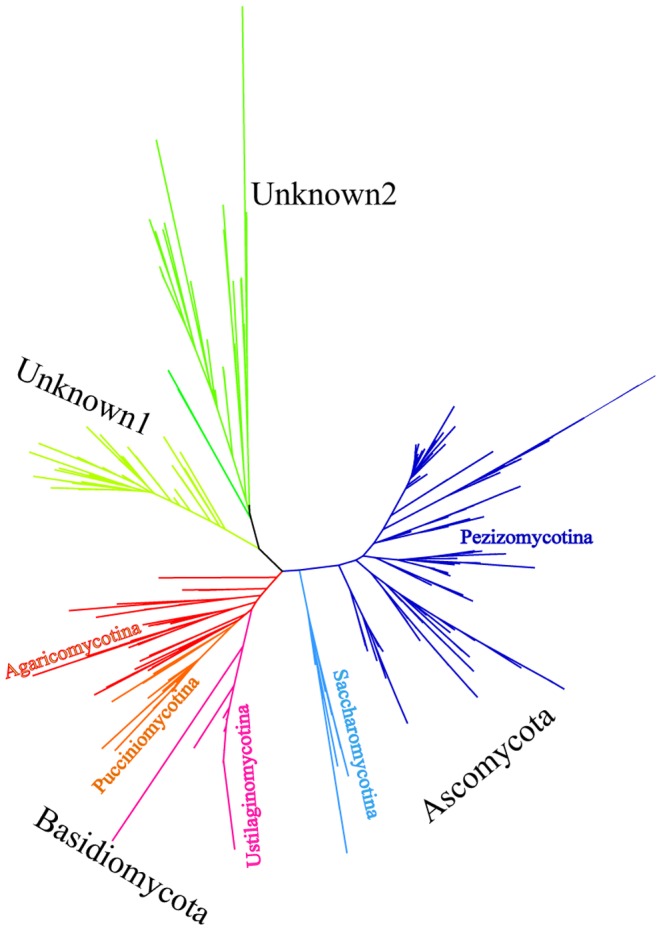
Maximum likelihood phylogenetic tree. 744 unique fungal sequences derived from 30 marine fungal clone libraries are aligned using Muscle v3.8 and undergo quality control in Mothur v1.25 before used for phylogenetic tree generation. Maximum likelihood calculation is done in PhyML v3.0 corrected by model HKY85. The best tree was returned by searching tree topology using subtree pruning and regrafting (SPR) algorithm starting with 5 random trees.

Phylogenetic analysis indicated that the majority of these fungal phylotypes belonged to two major phyla of Ascomycota and Basidiomycota with highly diversified fungal lineages within these individual phyla (multiple branches) (Figure 1). Similar findings were reported in a recent review in which the authors collected broad data sets of fungal small subunit ribosomal DNA (SSU rDNA) and concluded the abundance of these two phyla [22]. Members of the phylum Ascomycota was found to be affiliated with the subphyla Pezizomycotina and Saccharomycotina, whereas sequences of Basidiomycota belonged to three subphyla *i.e.* Agaricomycotina, Pucciniomycotina and Ustilaginomycotina. In addition to these two major phyla, two additional clades were not previously described in marine fungi (Figure 1 and Table S3 in File S1). These two clades were deeply branched. The majority of the OTUs had no affiliation to reported fungal sequences in the current database but had low sequence similarity to some eukaryotic sequences. Based on the current information, these sequences cannot be ruled out as fungal sequences and thus were included in the community analyses. This discovery thus indicates the presence of a possible largely unknown fungal community in the pelagic ocean whose roles in the microbial food web and biogeochemical cycles are yet to be discovered.

**Figure 2:**
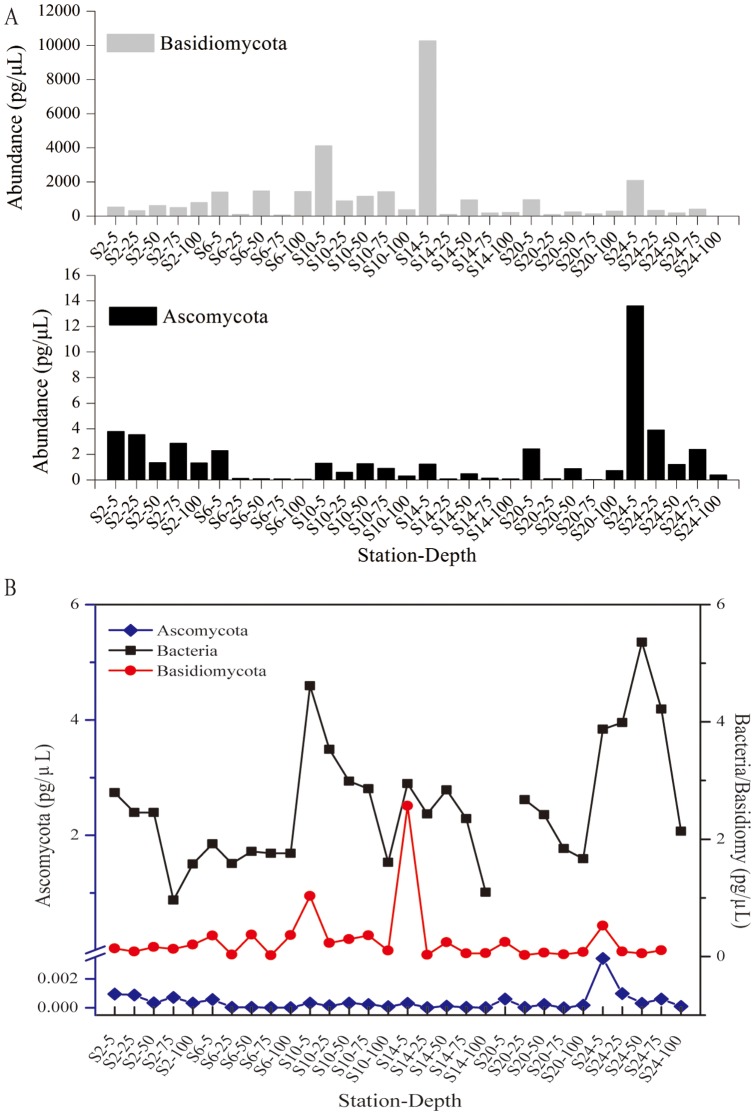
Evaluation of Ascomycota and Basidiomycota abundance by quantitative PCR among depths of all stations. The abundance represented by their total DNA concentration in the original sample was calculated based on standard curves created using two known Ascomycota and Basidiomycota species, respectively. Station 2 (N19/E-160) and 24 (N-36/E162) were coastal stations near Hawaii and Australia, respectively; Station 10 (N0/E-180) was station at the Equator; Station 6 (N10/E-170), 14 (N-9/E170) and 20 (N-25/E165) were open ocean stations.

Quantitative analysis (qPCR) indicated that the phyla Ascomycota was found to be most abundant in the coastal stations of the Pacific Ocean islands (Station 2/N19E-160 and Station 24/N-36E162) (p = 0.003), even though these two stations are separated by thousands of kilometers (Figure 2). Conversely, Basidiomycota was found to be abundant in both oceanic (station 10 & 14) and coastal (station 24) stations with highest abundance present at station 14 (equator region) (p = 0.587). Interestingly, Basidiomycota and bacterioplankton were in the similar order of magnitude of DNA quantity. Particularly, at the depth of 5m at the station 14, the abundance of Badisiomycota was similar to that of bacterioplankton (Figure 2B). Clearly, the abundance of Basidiomycota was much higher than that of Ascomycota in all stations (p = 0.005) (Figure 2). Our results clearly support that planktonic fungi play an important role in ocean nutrient cycling.

### Phylogenetic affiliation of fungal community

The majority OTUs (96.8%; 150 OTUs) of ascomycota belonged to the subphylum Pezizomycotina and only 5 of ascomycota OTUs derived from Australian coast (Station 24) were closely affiliated with the subphylum Saccharomycotina. OTUs of the subphylum Pezizomycotina belonged to 3 classes (Eurotinomycetes, Dothiodeomycetes, and Sordariomycetes) and affiliated to 10 different orders (Table S3 in File S1). The vast majority (91.2%; 52 OTUs) of the Eurotinomycetes OTUs were closely related to *Aspergillus* sp. or *Penicillium* sp. (99–100% similarity), and most of these fungal OTUs were obtained from two coastal stations (Station 2 & 24). The Dothiodeomycetes OTUs were affiliated with 3 fungal orders: Dothideales, Botryosphaeriales and Capnodiales, many of which had close affiliation with known fungal species such as *Hortaea werneckii* (15 OTUs), *Diplodia* sp. (10 OTUs), *Cladosprorium* sp. (15 OTUs) etc. (Table S3 in File S1). Finally, fungal OTUs of the class Sordariomycetes were members of 5 orders: Glomerellales, Hypocreales, Trichosphaeriales, Xylariales and Microascales. Majority of OTUs (67.8%) from this class were retrieved from coastal stations (Station 2 and 24) with the exception of 9 OTUs within the family of Nectriaceae that were mostly found in the open ocean stations (Table S3 in File S1).

Basidiomycota consists of 3 subphyla with the highest fungal diversity found in the subphylum Agaricomycotina (Table S3 in File S1). Many of these fungal OTUs were distantly related to known species (<90% sequence similarity), probably belonging to new species or new genera. For example, 3 fungal OTUs (S20D4-12/14/15) found in station 20 had less than 90% similarity to *Phlebia acanthocystis*. Twenty OTUs of the subphylum Pucciniomycotina had high similarity (close to 99%) to *Rhodotorula mucilaginosa*. In addition, all 66 OTUs of the subphylum Ustilaginomycotina were found to have the highest similarity to *Malassezia* sp. (86%–99% sequence similarity), indicating the abundance of *Malassezia* sp. in the open ocean. Interestingly, our previous study also revealed the abundance of this species in marine sponges [12], indicating the potential existence of both symbiotic and free-living forms of this fungal species.

The majority of OTUs found in the two unknown groups in the phylogenetic tree could not be attributed to any known fungi and only with low sequence similarity to other eukaryotic groups. More specifically, 122 OTUs of unknown group 1 were found to be affiliated (80–85%) with an unknown fungus sequence derived from marine subsurface sediments (Table S3 in File S1). OTUs of unknown group 2 (57 OTUs) were more diversified, belonging to unknown fungi or other eukaryotic sequences derived from marine environments. However, their identity could not be determined because they had low percentage of sequence coverage and low sequence similarity to other published sequences in the NCBI database. Out of 57 OTUs, 48 did not belong to any member of known fungi or other eukaryotes when comparing their full length sequences. In addition, the other 9 OTUs were distantly related to a known eukaryote sequences at the similarity level of around 80% (Table S3 in File S1). Clearly, results of this study suggest largely undiscovered fungal species in the oceanic water.

### Fungal community structure and diversity comparison

**Figure 3:**
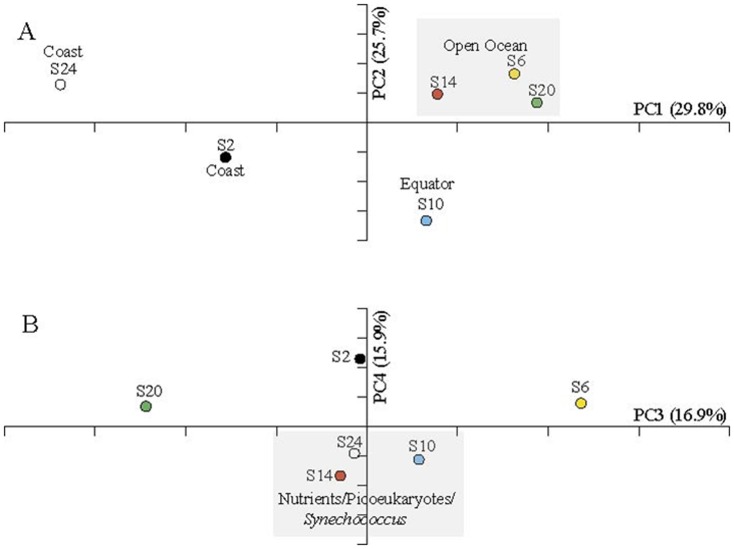
Principal Coordinates Analysis (PCoA) of marine fungal community based on the weighted-UniFrac distance matrix. Fungal communities from different locations were indicated as different symbols. The percentages of variation at the first four major coordinates were indicated. A. Fungal community variation explained by coordinates PC1 and PC2; B. Fungal community variation explained by coordinates PC3 and PC4.

**Figure 4:**
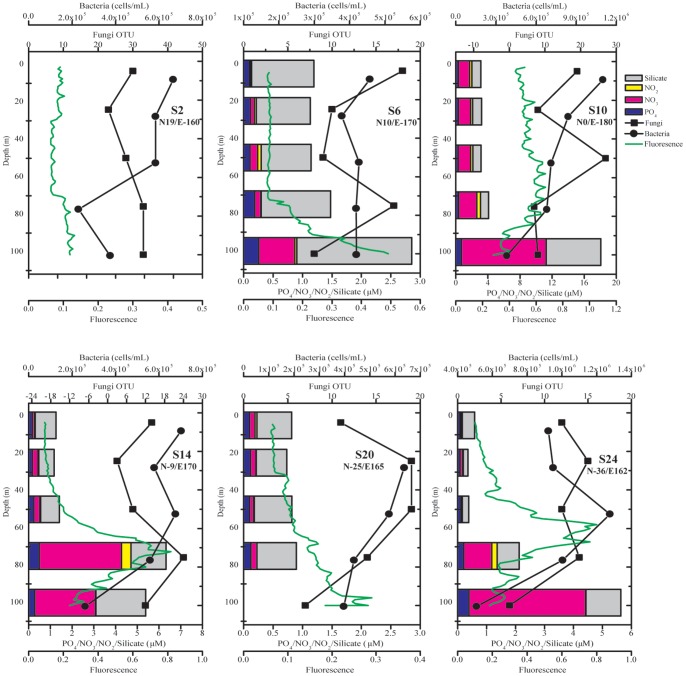
Marine fungal diversity along sampling stations. Fungal OTU number at various depths of each sampling station was plotted along with other biotic and abiotic factors. Fungal OTU (filled square), bacteria (filled circle), and chlorophyll (green line) were plotted against the sampling depths. At each depth, various nutrients including PO^4-^, NO^3-^, NO^2-^ and Silicic acid were indicated as colored bars.

The difference of fungal community was assessed using UniFrac, a phylogeny-based metric [23]. Overall, the fungal community displayed the highest diversity near Hawaii coast (Station 2/N19E-160) but similar among open ocean stations along the transect (Figure S2 in File S1). Results of this study supported that the higher mycoplankton diversity occurred in coastal waters than the open ocean and is in concordance with the previous report [5]. Carbon from autochthonous primary production and allochthonous (terrestrially derived) production is higher near the coast and this source that exceeds the consumption of herbivores, along with elevated levels of detritus and other forms of nutrients [24], favors increased mycoplankton abundance and diversity. This organic detritus serves as nutrient sources for fungal component in coastal marine ecosystems [25], making these environments a rich source of diverse mycoplankton communities. Similar findings were also reported in research of culturable fungi, in which fewer fungal isolates were obtained in pelagic water samples than those from coastal waters [26]. The unweighted UniFrac revealed an overall distance of 0.74 (p<0.001) between two communities of all stations. The fungal community comparison based on weighted UniFrac further indicated slightly higher distance within stations compared to that of between stations (Figure S3 in File S1), suggesting a complex vertical fungal community structure in the euphotic zone of specific locations. This was further supported by the hierarchical clustering where fungal communities from coastal stations (Stations 2 & 24) were readily grouped but fungi from other stations were dispersed along a nested structure (Figure S4 in File S1). The individual comparison between two fungal communities revealed overall high distances (Figure S5 in File S1), suggesting an underrated and versatile role of fungi in various marine environments. Weighted UniFrac-based principal coordinate analysis (PCoA) separated fungal communities primarily by locations where open ocean stations were clustered together with coastal and equator communities stood out (Figure 3A). This variation (PC1 of 29.8% and PC2 of 25.7%) by location was not readily explained by all measured environmental factors like nutrients or other autotrophs and heterotrophs. Compared to other bacterioplankton, fungi arm themselves with unique enzymatic system for hydrolyzing resistant long chain carbohydrate substrates [25]. A possible explanation for the variation thus could be their participation in the degradation of recalcitrant dissolved organic matters (RDOM) generated in the microbial carbon pump (MCP) [27]. However, further efforts on the elucidation of RDOM distribution and their degradation by fungi would be necessary to support this. The coordinates PC3 (16.9%) and PC4 (15.9%) further revealed variation reflected by nutrients (nitrate, nitrite and phosphate) level and other photosynthetic phytoplankton including picoeukaryotes and *Synechococcus* (Figure 3B and Figure S6 in File S1). This evidence thus suggests possible fungal consumption of dissolved organic matter (DOM) generated by these primary producers of ocean ecosystems and is also consistent with the report that the vertical distribution of fungal biomass closely related to that phytoplankton biomass in coastal water [21]. In this study, the three high nutrient stations (Station 10, 14 and 24) where nitrogen was not limited, the highest primary production was achieved at shallower depths; on the other hand, highest primary production in the oligotrophic stations (Station 6 and 20) was seen at deeper depths where nutrients were available (Figure 4). Overall, the primary producers including *Prochlorococcus*, *Synechococcus* and picoeukaryotes showed a similar vertical pattern to chlorophyll profile in both coastal and open ocean stations albeit the discrepancy that one might be slightly dominating the primary production than others at specific depths (Figure 4 and Figure S6 in File S1). As irreplaceable food web components, marine microbes reveal their importance in the carbon cycling in the pelagic zone of oceans [28], [29]. Heterotrophic bacteria and fungi share the function of mediating carbon and nutrients flux in marine ecosystem [30]. Among all stations, concentrations of bacteria were mostly higher near surface and decreases along depths, indicating their role in recycling and incorporating upwelling organic matter into higher food webs (Figure 4). Fungal OTUs followed a similar vertical pattern to bacteria in most stations, suggesting their involvement in the organic matter consumption (Figure 4). In addition, the abundance of Ascomycota and Basidiomycota at majority of the stations was relatively higher at surface and decreases along depths (Figure 2). Collectively, the fungal diversity and the abundance of major phyla revealed a possible involvement of fungi in organic matter consumption in the euphotic zone of ocean ecosystems.

All together, our results show that planktonic marine fungi are molecularly diverse, and that a variety of fungi could be participating in multiple biological processes including dissolved organic matter consumption. These results suggest that planktonic fungi are integral components of the marine microbial community, and are likely to be a versatile group involved in multiple biological processes and should be included in future marine microbial ecosystem models. Future multi-gene, genomic or transcriptomic analyses of fungal signatures of these open ocean ecosystems will help provide deeper insight into their molecular diversity and their functional role in pelagic ecology and biogeochemistry.

## Materials and Methods

### Sample collection

Seawater sample was collected from 5 depths (5 m, 25 m, 50 m, 75 m and 100 m) of 6 locations (Station 2 (N19.4942/E-160.0367), Station 6 (N10.0808/E-170.1366), Station 10 (N0.3653/E-179.644), Station 14 (N-9.2503/E169.9996), Station 20 (N-25.6717/E165.4164) and Station 24 (N-36.1654/E161.7915)) with the standard sampling rosette mounted around the CTD sensor package in the research cruise from Honolulu, Hawaii to Brisbane, Australia during January-February 2007 (Figure S1 in File S1). No specific permissions were required for these locations and activities. Vertical profiles of fluorescence were measured using a Turner Designs 10-AU fluorometer [31]. The concentration of bacteria, *Prochlorococcus*, *Synechococcus* and picoeukaryotes were measured using flow cytometry according to the methods described [32]. A modified Becton Dickinson FACSCalibur flow cytometer was used to quantitatively deliver samples. Cells were excited at 488 nm by a 15 mW Ar laser, and measured for forward scatter, size scatter, and fluorescence emissions (green (530±30 nm), orange (585±42 nm), red (>670 nm)). The characteristic flow cytometric signatures of bacteria, *Prochlorococcus*, *Synechococcus*, picoeukaryotic phytoplankton were applied in gating these populations following standard population gating schemes [33]. The water samples for measuring concentrations of PO_4_^-^, NO_3_^-^, NO_2_^-^, and silicate were collected in a trace metal clean rosette, filtered through 0.4 µm membranes and frozen at −20°C for further analysis. Dissolved PO_4_^-^, NO_3_^-^ and NO_2_^-^ concentrations were measured according to the methods described by Hynes et al [34]. Silicate was measured following the method described by Strickland and Parsons [35]. The field studies of this work did not involve endangered or protected species.

### DNA extraction and library construction

200 mL of each seawater sample was sequentially filtered through 2-micron and 0.22-micron polycarbonate filter membranes. The total genomic DNA was extracted from these two membranes using the FastDNA kit (Qbiogene, Irvine, CA) and was used as a PCR template for the amplification of the internal transcribed region (ITS) between the ribosomal RNA genes using the nested PCR approach following the procedure described by Gao et al. [12]. More specifically, a highly conserved fungal rRNA gene primer ITS1F and primer ITS4 [36] were used in the first round of PCR reaction, and 1 µL of the PCR product in the first round was used as the template DNA for the nested PCR performed using primer set ITS3 (5'-GCATCGATGAAGAACGCAGC-3') and ITS4 (5'-TCCTCCGCTTATTGATATGC-3'). All PCR reactions were performed following standard PCR protocol in 50 µL reactions for 35 cycles of denaturation at 95°C for 1 min, annealing at 55°C for 1 min and extension at 72°C for 1 min. PCR products from three separate amplification reactions were combined and purified using Gel and PCR Clean-Up Kit (Promega, Madison, WI). The purified PCR products were cloned into pGEM-T Easy vector (Promega, Madison, WI) and transformed into *E. coli* DH5α competent cells. Total of 30 (5 depths × 6 stations) ITS cloning libraries were constructed. For each library, plasmids carrying insert of correct size were sequenced using T7 primers. Sequences obtained in this study were deposited in GenBank under accession numbers of JX269176 - JX270134.

### Sequence analysis

Sequences obtained in this study were aligned using Muscle v3.8 [37]. The aligned sequences were imported into Mothur v1.25 [38] for further quality control, trimming to the same lengths for further diversity analysis. Sequence identifications were done using the NCBI BLAST and all sequences with 99% sequence identity were assigned into operational taxonomic units (OTUs) in Mothur [38]. Maximum likelihood calculation was done in PhyML v3.0 [39] corrected by model HKY85. The best tree was returned by searching tree topology using subtree pruning and regrafting (SPR) algorithm with 5 random starting trees.

### Diversity comparison

The diversity within each fungal community (alpha diversity) was assessed by plotting rarefaction curve or calculating inverse Simpson index [38]. The similarity comparison between two communities (beta diversity) was performed based on UniFrac metric [23]. UniFrac distances are calculated based on the fraction of branch length shared between two communities in a phylogenetic tree constructed for all compared fungal communities. Mainly weighted Unifrac was employed in this study to assess the structure (membership and abundance) of fungal community. Principle coordinates analysis (PCoA) based on Unifrac distances was employed [38] to determine the major factors that separate fungal communities. The overall diversity comparison among all 30 fungal communities (5 depths × 6 stations) was generated into a heatmap based on the weighted UniFrac distances [38]. Hierarchical clustering was conducted using Unifrac distances based on the unweighted pair group method with arithmetic mean (UPGMA).

### Quantitative PCR

Quantitative PCR was employed to assess the abundance of two major phyla of Ascomycota and Basidiomycota in all sampling sites. The fungal abundance was calculated by comparing to a standard curve plotted with cycle threshold (Ct) against DNA concentration. The concentration gradients of 0.01, 0.1, 1, 10, 100 µg/µL from the known Ascomycota and Basidiomycota DNA were applied for generating the standard curve. Ascomycota and Basidiomycota DNA were amplified using the highly conserved fungal rRNA gene primers (ITS1F, ITS4-Asco and ITS4-Basidio) [40]. PCR reactions (20 µL) contained 10 µL of KAPA SYBR FAST Universal 2X qPCR Master Mix (Kapa Biosystems), 1 µL of template DNA, 0.5 µL of 10 µM ITS1F, 0.5 µL of 10 µM ITS4-Asco or ITS4-Basidio, and 8 µL of water. The qPCR protocol was set up with an initial 3 min denaturation at 95°C, followed by 40 amplification cycles of 95°C for 1 min, 55°C for 45 s, and 72°C for 1 min. All PCR reactions were done in triplicate. The statistical significance (p<0.05) of Ascomycota and Basidiomycota abundance comparison between stations was determined by one-way analysis of variance (ANOVA) using untransformed data. The paired t-test (p<0.05) was performed to determine the difference between the Ascomycota and Basidiomycota abundance within each station.

## Supporting Information

File S1 (DOC)
