# Supplementary material for: Distribution and Diversity of Planktonic Fungi in the West Pacific Warm Pool
Source: PLoS One. 2014 Jul 3;9(7):e101523. doi: 10.1371/journal.pone.0101523 (PMC4081592; doi:10.1371/journal.pone.0101523)
Supplement: File S1 — (DOC) [file pone.0101523.s001.doc]

Supplementary Information for

**Distribution and Diversity of Planktonic Fungi in the West Pacific Warm Pool**

Xin Wang, Purnima Singh, Zheng Gao, Xiaobo Zhang, Zackary I. Johnson, and Guangyi Wang^*^

*To whom correspondence should be addressed.

Email: [gywang@tju.edu.cn/guangyi@hawaii.edu](mailto:gywang@tju.edu.cn/guangyi@hawaii.edu)

**Supplemental Figure Legends**

**Figure S1.** **CTD sampling stations in the Western Pacific during the cruise of R/V Kilo Manoa in June 2008.** Station locations were indicated as the number in the white circle.

**Figure S2.** **Rarefaction curve for marine fungal diversity among 6 stations.** Phylogenetic diversity is represented by branch length.

**Figure S3.** **Mean (±SEM) weighted UniFrac distance of fungal communities within and between various stations.**

**Figure S4.** **Hierarchical clustering of fungal communities in various stations and depths.** The bar indicates a weighted UniFrac distance of 0.1.

**Figure S5.** **Heatmap showing the fungal diversity comparison among various stations and depths.** The scale at the bottom of the heatmap indicates the similarity level between each comparison. The darker the color is, the more different the two comparing fungal communities are.

**Figure S6.** **Distribution of various nutrients (PO_4_^-^, NO_3_^-^, NO_2_^-^ and Silicate), picoeukaryotes and *Synechococcus* among investigated sampling locations.** For each station, the data was averaged from the measurements of 5 different depths representing the station abundance.

Figure S1


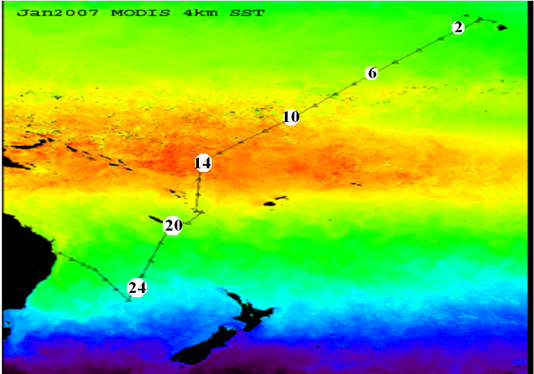


Figure S2


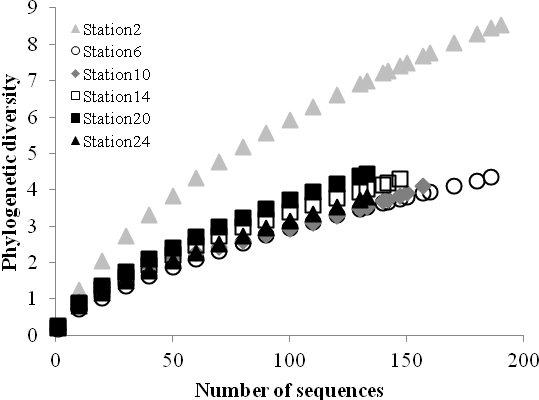


Figure S3


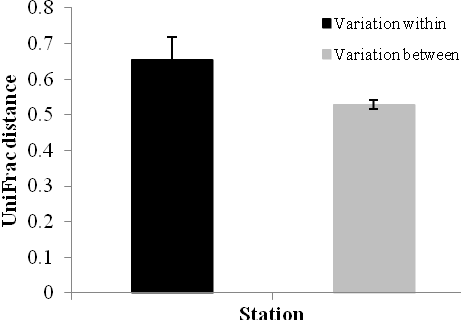


Figure S4


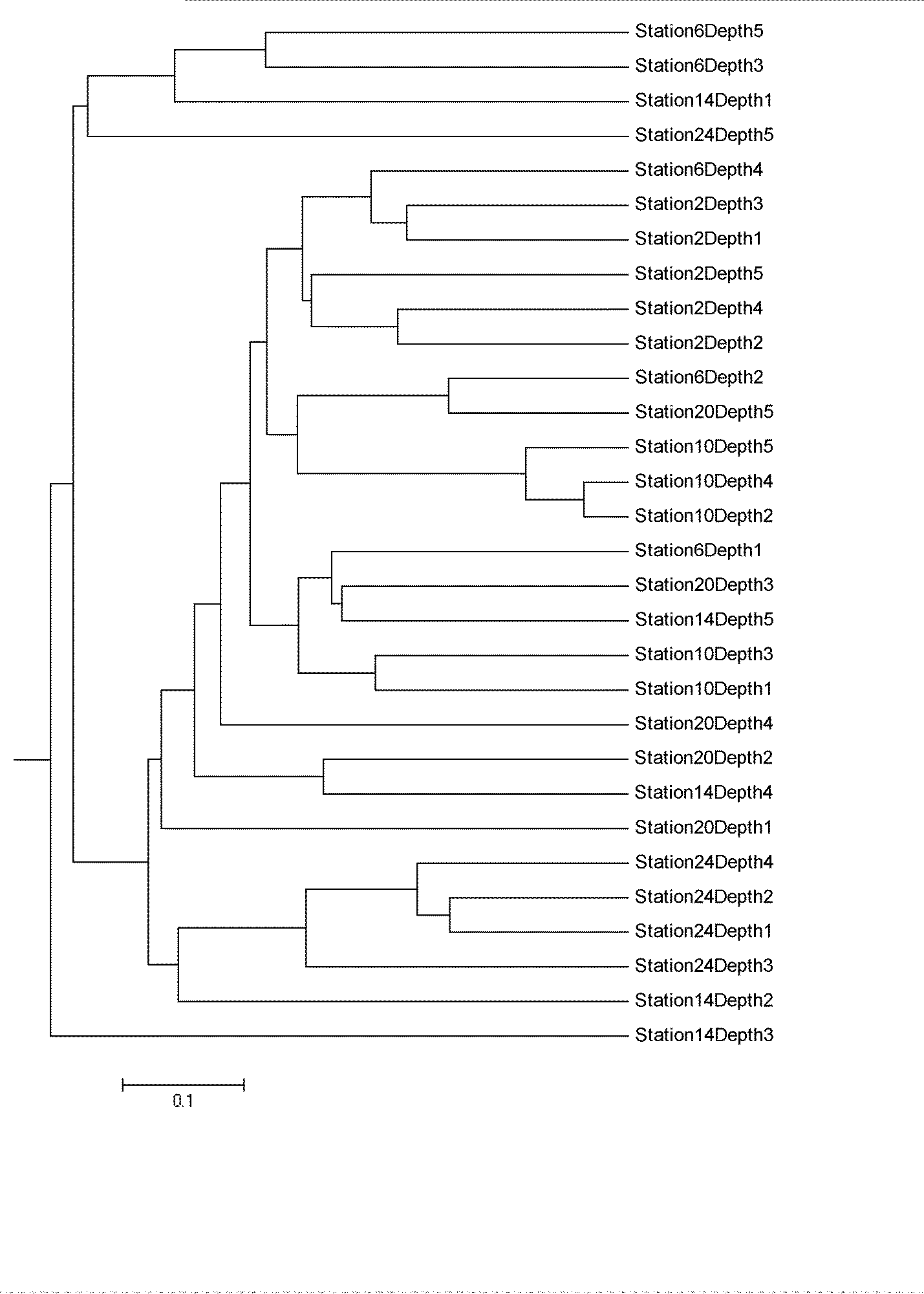


Figure S5


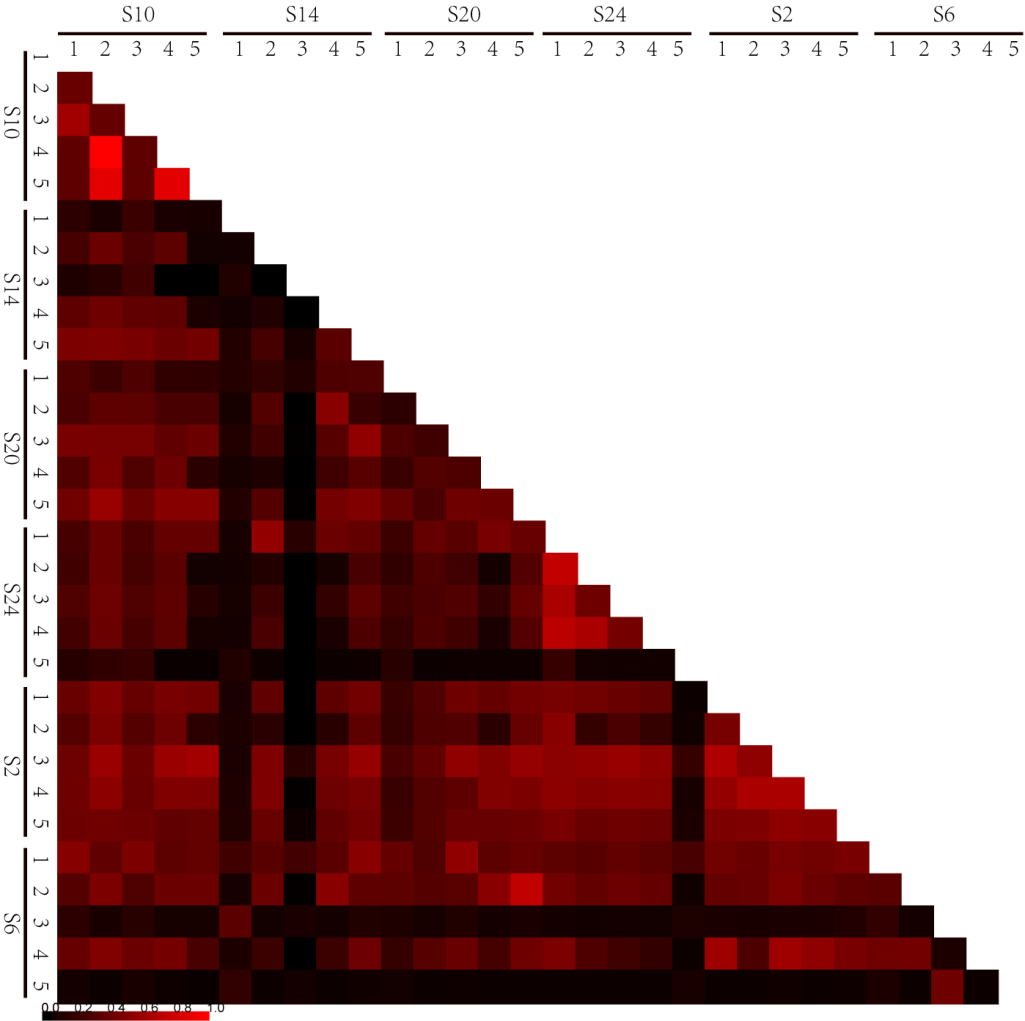


Figure S6


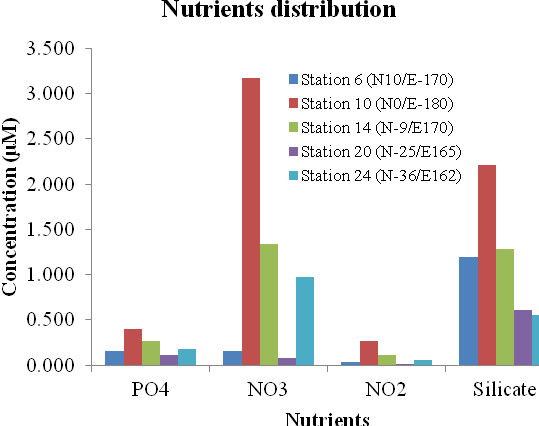


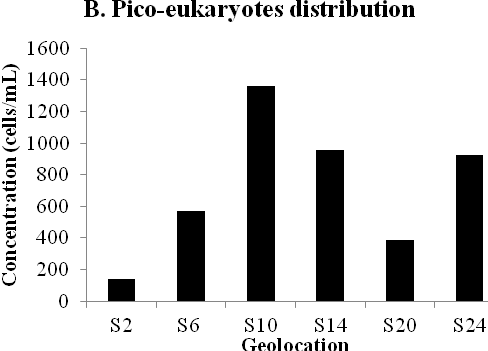


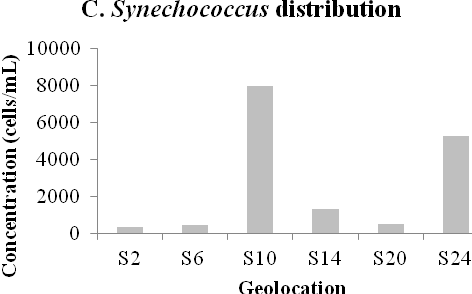


**Table S1** Diversity indices for fungal communities from 5 depths of 6 different stations. For each fungal community, the sequencing numbers (Seqs#) and the obtained OTU number (OTUs) are recorded. The coverage of each library is calculated and the Inverse Simpson Index is calculated to indicate the estimated fungal diversity from each community.

| Group | Seqs# | Coverage | OTUs | Inverse Simpson Index |
| --- | --- | --- | --- | --- |
| Station2Depth1 | 34 | 0.205882 | 30 | 112.2 |
| Station2Depth2 | 27 | 0.259259 | 23 | 70.2 |
| Station2Depth3 | 34 | 0.264706 | 28 | 46.75 |
| Station2Depth4 | 46 | 0.413043 | 33 | 39.80769 |
| Station2Depth5 | 53 | 0.566038 | 33 | 33.60976 |
| Station6Depth1 | 25 | 0.36 | 18 | 18.75 |
| Station6Depth2 | 39 | 0.923077 | 10 | 5.110345 |
| Station6Depth3 | 42 | 0.857143 | 9 | 2.979239 |
| Station6Depth4 | 37 | 0.675676 | 17 | 8.222222 |
| Station6Depth5 | 43 | 0.883721 | 8 | 3.778243 |
| Station10Depth1 | 29 | 0.448276 | 19 | 12.6875 |
| Station10Depth2 | 37 | 0.837838 | 8 | 1.748031 |
| Station10Depth3 | 39 | 0.487179 | 27 | 35.28571 |
| Station10Depth4 | 39 | 0.871795 | 7 | 1.490946 |
| Station10Depth5 | 13 | 0.538462 | 8 | 7.090909 |
| Station14Depth1 | 44 | 0.772727 | 14 | 2.657303 |
| Station14Depth2 | 40 | 1 | 3 | 2.215909 |
| Station14Depth3 | 9 | 0.222222 | 8 | 36 |
| Station14Depth4 | 39 | 0.538462 | 24 | 21.79412 |
| Station14Depth5 | 15 | 0.333333 | 12 | 26.25 |
| Station20Depth1 | 18 | 0.5 | 11 | 9.5625 |
| Station20Depth2 | 57 | 0.754386 | 19 | 3.883212 |
| Station20Depth3 | 23 | 0.26087 | 19 | 36.14286 |
| Station20Depth4 | 35 | 0.771429 | 14 | 6.134021 |
| Station20Depth5 | 9 | 0.333333 | 7 | 12 |
| Station24Depth1 | 14 | 0.214286 | 12 | 30.33333 |
| Station24Depth2 | 37 | 0.810811 | 15 | 11.28814 |
| Station24Depth3 | 14 | 0.214286 | 12 | 30.33333 |
| Station24Depth4 | 27 | 0.62963 | 14 | 7.02 |
| Station24Depth5 | 41 | 0.926829 | 6 | 3.203125 |

**Table S2** Diversity indices for fungal communities from 6 sampling stations. Fungal data from 5 depths of each station were combined and treated as one large fungal community. For each fungal community, the sequencing numbers (Seqs#) and the obtained OTU number (OTUs) are recorded. The coverage of each library is calculated and the Inverse Simpson Index is calculated to indicate the estimated fungal diversity from each community.

| Group | Seqs# | Coverage | OTUs | Inverse Simpson Index |
| --- | --- | --- | --- | --- |
| Station2 | 194 | 0.463918 | 131 | 89.14762 |
| Station6 | 186 | 0.784946 | 54 | 11.34146 |
| Station10 | 157 | 0.757962 | 53 | 4.619389 |
| Station14 | 147 | 0.693878 | 60 | 14.28895 |
| Station20 | 142 | 0.683099 | 64 | 18.33517 |
| Station24 | 133 | 0.75188 | 49 | 14.90323 |

**Table S3** Phylogenetic affiliation of marine fungi.

| Phylum | Sub-phylum | Class | Order | Family | Representative Species  (accession no.) | OTU number  (accession no. of representative OTU) | Major distribution |
| --- | --- | --- | --- | --- | --- | --- | --- |
| Ascomycota | Pezizomycotina | Eurotiomycetes | Eurotiales | Trichocomaceae | *Aspergillus* sp. (JQ717355) | 52  (JX269320) | S2/24 (coast) |
|  |  |  |  |  | *Penicillium* sp. (JN252117) |  |  |
|  |  |  | Chaetothyriales | Herpotrichiellaceae | *Exophiala dermatitidis* (GQ911541) | 1  (JX269558) | S10 |
|  |  |  |  | Mitosporic Chaetothyriales | *Sarcinomyces* sp.  (AJ972813) | 4  (JX269226) | S2 |
|  |  | Dothideomycetes | Dothideales | Dothioraceae | *Aureobasidium* sp. (JQ235064) | 5  (JX269853) | S2 |
|  |  |  |  | Mitosporic Dothideales | *Hortaea werneckii* (JX141367) | 15  (JX269216) | S2 |
|  |  |  | Botryosphaeriales | Botryosphaeriaceae | *Diplodia* sp. (EU860397) | 10  (JX269308) | S2 |
|  |  |  | Capnodiales | Mycosphaerellaceae | *Pseudocercosporella fraxini* (GU214682) | 1  (JX270041) | S24 |
|  |  |  |  | Davidiellaceae | *Cladosporium* sp. (JN851005) | 15  (JX269358) | S2/14/20 |
|  |  |  | Not determined | | *Epicoccum nigrum* (GQ996573) | 19  (JX269241) | S2/6/24 |
|  |  | Sordariomycetes | Glomerellales | Plectosphaerellaceae | *Acrostalagmus* sp. (GU813970) | 6  (JX269207) | S2 |
|  |  |  | Hypocreales | Mitosporic Hypocreales | *Trichothecium* sp. (EU622273) | 1  (JX270005) | S24 |
|  |  |  |  | Nectriaceae | *Gliocladiopsis* sp. (FJ481051) | 9  (JX269402) | S6/10/14/20  (open ocean) |
|  |  |  | Trichosphaeriales | Mitosporic Trichosphaeriales | *Nigrospora* sp. (FJ904917) | 8  (JX269213) | S2 |
|  |  |  | Xylariales | Amphisphaeriaceae | *Pestalotiopsis* sp. (JX305729) | 3  (JX269352) | S2 |
|  |  |  | Microascales | Halosphaeriaceae | *Periconia* sp. (HQ607981) | 1  (JX269210) | S2 |
|  | Saccharo-mycotina | Saccharo-mycetes | Saccharomycetales | Saccharomycetaceae | *Debaryomyces* sp.  (JQ912667) | 5  (JX270054) | S24 |
|  | Mitosporic ascomycota | | | | *Scolecobasidium* sp.  (FJ914794)  *Ochroconis*sp. (AB161066) | 9  (JX269201) | S2/6/20 |
| Basidiomycota | Ustilagino-mycotina | Exobasidio-mycetes | Malasseziales | Malasseziaceae | *Malassezia* sp. (GU327510) | 66  (JX269243) | S2/10 |
|  | Pucciniomycotina | Microbotryo-mycetes | Sporidiobolales | mitosporic Sporidiobolales | *Rhodotorula mucilaginosa* (JX156378) | 20  (JX269838) | S6/14/20 |
|  |  | Cystobasi-diomycetes | Erythrobasidiales | Erythrobasidiaceae | *Sporobolomyces* sp.  (JQ936286) | 1  (JX269238) | S2 |
|  | Agaricomycotina | Tremellomycetes | Filobasidiales | mitosporic Filobasidiales | *Cryptococcus* sp. (JQ993379) | 2  (JX269393) | S6/14 |
|  |  |  | Polyporales | Polyporaceae | *Microporus* sp. (JQ409362) | 1  (JX269992) | S20 |
|  |  |  | Corticiales | Corticiaceae | *Phlebia* sp. (AB210075) | 4  (JX269967) | S20 |
|  |  |  | Agaricales | Marasmiaceae | *Marasmius* sp. (HQ248212) | 5  (JX269298) | S2/6/20 |
|  |  |  | Hymenochaetales | Hymenochaetaceae | *Phellinus* sp. (AY558610) | 7  (JX269810) | S14 |
|  |  |  |  |  | *Fuscoporia gilva* (AY558620) | 2  (JX269494) | S6 |
| Unknown 1 | *Incertae sedis* | | | | Uncultured fungus/eukaryote  (AB615504)  (no known fungal affiliation and low sequence similarity to eukaryotes) | 122  (JX269239) | All stations |
| Unknown  2 | *Incertae sedis* | | | | No known affiliation with full length sequence alignment  (partial aligned to eukaryotes) | 48  (JX269816) | S10/14/20 (open ocean) |
|  | *Incertae sedis* | | | | Uncultured eukaryote clone  (GU942406) | 9  (JX269237) | S2/6/10 |
